# Exploring Engagement With and Effectiveness of Digital Mental Health Interventions in Young People of Different Ethnicities: Systematic Review

**DOI:** 10.2196/68544

**Published:** 2025-04-07

**Authors:** Rinad Bakhti, Harmani Daler, Hephzibah Ogunro, Steven Hope, Dougal Hargreaves, Dasha Nicholls

**Affiliations:** 1 Department of Brain Sciences Division of Psychiatry Imperial College London London United Kingdom; 2 University of Lancaster Lancaster United Kingdom; 3 Imperial College Healthcare NHS Trust London United Kingdom; 4 School of Public Health Imperial College London London United Kingdom

**Keywords:** digital mental health interventions, young people, ethnicity, engagement, effectiveness, artificial intelligence, AI

## Abstract

**Background:**

The prevalence of mental health difficulties among young people has risen in recent years, with 75% of mental disorders emerging before the age of 24 years. The identification and treatment of mental health issues earlier in life improves later-life outcomes. The COVID-19 pandemic spurred the growth of digital mental health interventions (DMHIs), which offer accessible support. However, young people of different ethnicities face barriers to DMHIs, such as socioeconomic disadvantage and cultural stigma.

**Objective:**

This review aimed to summarize and evaluate the engagement with and effectiveness of DMHIs among young people of different ethnicities.

**Methods:**

A systematic search was conducted in MEDLINE, Embase, and PsycINFO for studies published between January 2019 and May 2024, with an update in September 2024. The inclusion criteria were participants aged <25 years using DMHIs from various ethnic backgrounds. Three reviewers independently screened and selected the studies. Data on engagement (eg, use and uptake) and effectiveness (eg, clinical outcomes and symptom improvement) were extracted and synthesized to compare findings. Studies were assessed for quality using the Mixed Methods Appraisal Tool.

**Results:**

The final search yielded 67 studies, of which 7 (10%) met inclusion criteria. There were 1853 participants across the 7 studies, all from high-income countries. Participants were predominantly aged 12 to 25 years, with representation of diverse ethnic identities, including Black, Asian, Hispanic, mixed race, and Aboriginal individuals. Engagement outcomes varied, with culturally relatable, low-cost interventions showing higher retention and user satisfaction. Linguistic barriers and country of origin impeded the effectiveness of some interventions, while near-peer mentorship, coproduction, and tailored content improved the effectiveness of DMHIs. While initial results are promising, small sample sizes, heterogeneity in outcome assessments, and a paucity of longitudinal data impeded robust comparisons and generalizability.

**Conclusions:**

DMHIs show potential as engaging and effective mental health promotional tools for young people of different ethnicities, especially when coproduced and culturally relatable. Initial data suggest that interventions facilitating near-peer mentoring, linguistic adaptation, low cost, and cultural relatability have improved engagement and effectiveness. Future research should focus on developing a consensus definition of DMHIs, exploring DMHIs in children aged <12 years, and conducting detailed qualitative and quantitative research on use factors and treatment efficacy of DMHIs for young people of different ethnicities.

**Trial Registration:**

PROSPERO CRD42024544364; https://tinyurl.com/yk5jt8yk

## Introduction

### Background

The mental health of children and young people has been a continuing topic of interest in global health care, with 75% of mental health issues occurring before the age of 24 years [1]. The COVID-19 pandemic has further exacerbated the prevalence of mood and sleep symptoms and disrupted education and social interaction among youth [2]. Due to these increased needs, mental health services globally are stretched to their capacity, raising concerns regarding their ability to meet this need [3].

Rapid identification and management of young people with mental health conditions can significantly improve well-being and socioeconomic outcomes later in life [4,5]. However, ethnic minority youth tend to be less likely to seek and receive mental health care, despite controlling for the presence of symptomatology and psychiatric diagnoses [6,7]. Suggested causes for this disparity in treatment for ethnic minority youth include financial concerns, lack of time, and stigma [8]. While these factors have been identified, research in the field of youth mental health within ethnic minority groups continues to be sparse, and ethnic inequalities have continued to widen [9,10].

Given young people’s comfort and acceptance of technology and the potential for school and university campuses to address disparities in health care earlier in life, digital mental health interventions (DMHIs) offer an enticing solution to addressing these barriers [11]. In recent years, DMHI have rapidly grown in use and development, particularly during and, in part, due to the COVID-19 pandemic [12]. Several interventions have been shown to improve symptoms of anxiety and depression, and the convenience of digitally delivered treatment offers economic benefits to both patients and resource-stretched health care systems [12,13].

While promising, research into DMHI has grappled with variability in terms of user engagement and uptake [14]. Several studies have attempted to explore the factors underpinning poor uptake, suggesting that the factors promoting initial use may differ from those that promote continued engagement [15]. Most of the current literature underscores methods of promoting user reengagement, but initial use remains poorly understood and has been identified as a research gap in the recent World Health Organization (WHO) guidelines [9]. Brouwer et al [16] found that user-level factors, such as personal motivations and perception of a digital intervention, may be implicated in the process of initial user engagement. Hence, it is important to also study the experiences of young people of different ethnicities in improving the current understanding of the factors affecting initial uptake and continuing the use of DMHI, such that the interventions may be more effective in addressing ethnic minority barriers to support health care.

### This Review

This review aimed to summarize and evaluate the engagement of young people of different ethnicities with DMHI and the effectiveness of DMHI in these populations. In this review, we defined DMHI as digitally delivered products designed for the preventative, diagnostic, therapeutic, or psychological benefit of an individual’s mental health [14-16]. These ranged from websites and virtually delivered care to video games and mobile apps relating to mental health [15,16]. We defined engagement as the extent to which and how individuals perceive and interact with a DMHI, remain invested in the experience of a DMHI, and integrate the DMHI into their lives. Therefore, the effectiveness of a DMHI refers to the extent to which it achieves its intended outcomes, such as improvements in symptom scoring, clinical outcomes, behavior, or well-being after a user has engaged with the intervention. Studies using both quantitative and qualitative measures were examined to assess engagement and effectiveness across different ethnic groups. Through this work, researchers may be further informed on methods of improving the feasibility and acceptability of DMHI in an underserved population group.

## Methods

### Overview

Established PRISMA (Preferred Reporting Items for Systematic Reviews and Meta-Analyses) guidelines were used to inform the process of this systematic review, both in the identification and analysis of relevant studies [17]. This systematic review was registered in PROSPERO of the National Institute for Health Research (CRD42024544364). Ethics approval was not sought for this study as it was an evidence synthesis of existing published research.

### Databases and Information Sources

Initial searches to identify relevant subject headings and keywords were conducted across three databases—MEDLINE, Embase, and PsycINFO—in May 2024, evaluating publications in peer-reviewed journals from January 2019 to May 2024. An additional updated search was conducted in September 2024. These databases were selected based on relevance to the field of research and familiarity with the search strategy (Multimedia Appendix 1). The review focused on the last 5 years, as DMHIs have seen significant growth in development and use during this period, accelerated by the COVID-19 pandemic [12].

The search terms explored were “Mental Health,” “Mental Disorders,” “Psych*,” “Computer-Assisted Therapy,” “Internet Based Intervention,” “Mental Health Teletherapy,” “Digital Mental Health,” “Digital Intervention,” “Online Therap*,” “eTherap*,” “Web-based Intervention,” “e-Mental Health,” “Mental Health Mobile Applications,” “Adolescent,” “Child,” “Student,” “Attitude*,” “Experience*,” “Engagement,” “Respons*,” “Ethnic and Racial Minorities,” and “Ethnic*.”

These search terms were mapped using population, intervention, control, and outcome (Textbox 1) and checked through consultation with a medical librarian. These terms were then combined using Boolean operators and applied to a search.

**Eligibility criteria and the population, intervention, control, and outcome framework.**

**Inclusion criteria**

- Population: young people aged <25 years
- Intervention: digital mental health intervention
- Control: all primary research studies
- Outcome: experience of young people of different ethnicities using digital mental health interventions, including engagement and effectiveness of these interventions within these populations; both quantitative and qualitative measures of outcome
- Setting: any country
- Publication: available in English and published after 2019

**Exclusion criteria**

- Population: young people aged >25 years
- Intervention: interventions not addressing mental health and nondigital interventions
- Control: abstracts, posters, editorials, letters, dissertations, or conference presentations
- Outcome: lack of reporting on experience of young people with digital mental health interventions by ethnicity
- Setting: none
- Publication: not available in English and published before 2019

### Eligibility Criteria and Selection Process

The inclusion criteria for relevant articles were as follows: (1) published between 2019 and 2024; (2) published in an English language, peer-reviewed journal; (3) participants aged ≤25 years; and (4) reporting on ethnic or racial minority differences and perspectives in the experience and use of DMHI. Exclusion criteria were as follows: (1) not enough data or analysis of ethnic or racial differences, (2) study focused on practitioner or parent perspectives, and (3) the study population included adults aged >25 years. Gray literature, opinion articles, and case studies were excluded.

Subsequently, we manually screened the identified studies for duplicates and removed them. Three reviewers then independently evaluated the titles and abstracts of identified articles against the eligibility criteria. These reviewers also evaluated the full text of the remaining eligible articles after abstract and title screening, and any discrepancies in included or excluded studies between reviewers were resolved through discussion.

### Data Items

The data extracted outlined the first author, publication date, location, sample size, type of study, age, ethnic demographics, psychological measures or research tools used, DMHI used, and relevant outcomes (eg, perceived efficacy and psychological measure scoring) to the scope of this review. Missing or unclear data were not requested from or discussed with the authors of included papers, and reviewers resolved any discrepancies in the included data through discussion.

### Data Synthesis

Data were synthesized and presented in a table format for clarity. The results were interpreted by grouping them based on common themes or patterns identified across studies as well as highlighting key findings across individual studies. Differences or inconsistencies not replicated across studies were explored in the context of sample characteristics, study design, and other relevant factors.

### Quality Assessment

The quality assessment was conducted using the Mixed Methods Appraisal Tool (MMAT) from McGill University [18], due to the broad spectrum of methodologies used by the included studies. The MMAT evaluates the quality of qualitative, quantitative, and mixed methods studies. It does not provide an overall numeric score but instead assesses studies based on 5 methodological criteria specific to their design, classifying them as high, moderate, or low quality based on the number of criteria met. Two reviewers independently assessed each included study against the framework, and any disagreements in quality assessment were resolved through discussion. All 7 studies in this review were rated as having met the criteria outlined in the MMAT checklist.

## Results

### Overview

**Figure 1:**
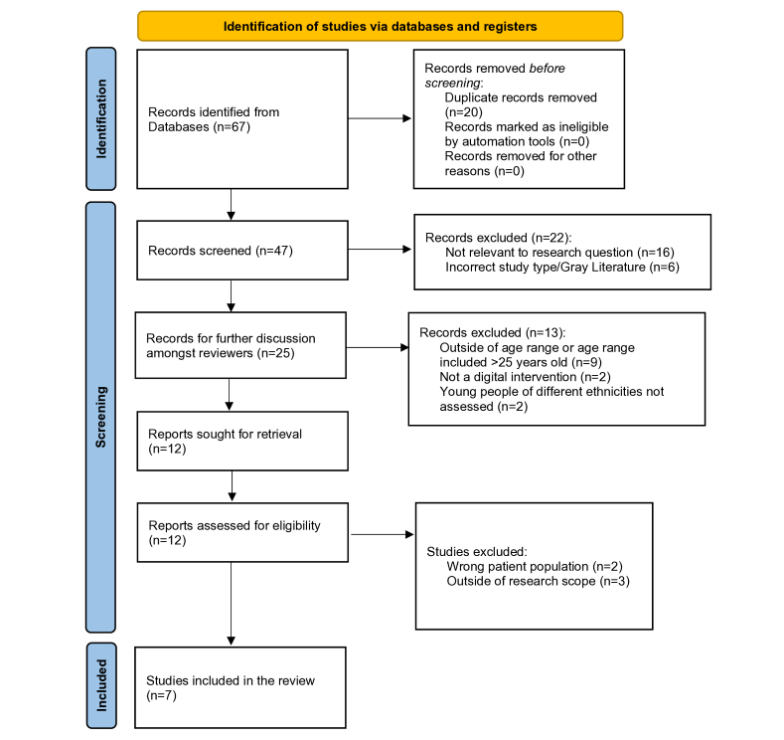
PRISMA diagram outlining the study selection.

The initial search conducted in May 2024 yielded 67 studies, with the study selection process outlined in the PRISMA workflow in Figure 1 (the PRISMA checklist is available in Multimedia Appendix 2 [17]). We identified 6 eligible studies in our initial search. An updated search conducted in September 2024 yielded an additional study, increasing the total to 7 studies. Table 1 contains detailed data on the 7 included studies regarding demographics, sample sizes, digital interventions used, and the outcomes measured that were relevant to our research question (measures of user engagement and effectiveness of the DMHI). Three studies primarily examined engagement [19-21], while 3 others addressed effectiveness [22-24], and 1 study [25] explored both engagement and effectiveness. The studies were all from high-income countries and conducted in English, and all the included studies achieved the maximal quality assessment rating according to the MMAT.

**Table 1:** Data extraction table, individually outlining key data from the included studies.

Study	Location; type of study; sample size	Age; ethnic and racial demographics	Research tools used	Digital intervention used	Reported outcomes (*P* values where applicable)
Ahuvia et al [19], 2022	United States; cross-sectional survey; 1224	95% of the participants were aged between 18 and 25 years; 5% were aged >25 years; White, non-Hispanic: 40%; Asian, non-Hispanic: 38.4%; Hispanic: 13%; Black, non-Hispanic: 3.3%; and others: 5.2%	Online student interest questionnaire; GAD-7^a^; PHQ^b^-9	Teletherapy and online self-help	Assessed only factors influencing interest and engagement, not effectiveness of any interventions. Interest in free teletherapy: Students of color: 73% White, non-Hispanic: 70% Interest in free self-help: Students of color: 72% White, non-Hispanic: 68% Interest in at-cost teletherapy: Students of color: 15% White, non-Hispanic: 27% Interest in at-cost self-help: Students of color: 22% White, non-Hispanic: 29% Significant finding: Asian students had significantly lower interest in at-cost teletherapy (P<.001) compared to White, non-Hispanic students.
Giovanelli et al [25], 2023	United States; mixed methods; 14	12-18 years; White: 64%; Asian: 14%; Black: 14%; Mixed race: 7%	PHQ-8; GAD-7; Youth Top Problems Assessment; MYAS^c^; Satisfaction with the program (Net promoter score); Qualitative: semistructured interviews with participants	Appa Health: smartphone app; web-based near-peer mentorship (pairing based on sociodemographics); CBT^d^ video content	Assessed both engagement and effectiveness. Engagement: Limited sample size resulted in limited subgroup analysis by ethnicity—only qualitative responses suggesting that the choice of mentor by ethnicity or similar heritage was positively received Median of 107 minutes over a 12-week period spent in video calls between mentors and mentees Short-form CBT videos provided weekly; 100% of participants expressed positive responses All participants completed the 12-week program and surveys, showing strong program retention and sustained engagement Net promoter score=85.75/100 Participants highlighted app accessibility and ease of use Effectiveness (depression and anxiety scoring after DMHI^e^ use): Median reduction in PHQ-8 scores: 3.5 points Median reduction in GAD-7 scores: 2.0 points Youth top problems (performance and interpersonal concerns): Mentoring experience Mean MYAS score 35.1 (out of 36) 100% of participants found mentoring beneficial Participants were enthusiastic about Appa, citing the reduction of barriers to care, highly positive mentor experiences, and appreciation of the short-form videos. Coproduction and cultural relatability work: Intervention developed with input from youth and clinical advisory boards Youth advisors provided feedback on app content for relatability and engagement, and mental health professionals ensured clinical accuracy Mentors selected for relatable lived experiences from a diverse pool of backgrounds Video content designed in short-form format, ranging from 30 to 90 seconds in duration Participant feedback loops and ability to customize mentor pairing based on identity preferences
Syed Sheriff et al [22], 2023	United Kingdom; randomized controlled trial; 463	16-24 years; White (British, Irish, or other): 78%; Asian British (Indian, Pakistani, or Bangladeshi): 7.1%; Black or Black British (Caribbean, African, or other): 4.8%; Chinese or Chinese British: 2.2%; mixed race (other): 3.7%; mixed race (White and Black or Black British): 3.2%; other or prefer not to say: 1.3%	PANAS^f^; K10^g^; Gorilla Experiment Builder; Feedback questionnaires	Web-based cultural experience; online website	Predominantly assessed effectiveness in young people of different ethnicities, with general nonspecific assessment of engagement. Engagement: Limited quantitative analysis due to security; 75% of participants completed all follow-up assessments, indicating a high retention rate Content exhaustion—participants felt they had exhausted all available content before the end of the intervention phase Effectiveness assessment in young people of different ethnicities: Significant differences in ethnic minority groups after culturally adapted intervention (−0.45 treatment effect on negative affect, 95% CI−0.6 to −0.2) Mean K10 and negative affect improvements population-wide using either cultural experience or standard website Cultural relevance and coproduction: WOB^h^ coproduced over a 3-month period, using an iterative process Input from key stakeholders in the development of WOB–young people aged 16-24 years, museum curators, youth engagement officers, and education officers Stakeholders involved in the selection of stories, creation of viewpoints, and determination of audio-visual preferences. Commenting tool incorporated into WOB Accessible language–simple English used
Routledge et al [21], 2022	Australia; mixed methods; 235	12-14 years; Non-Indigenous: 91.9%; Aboriginal or Torres Strait Islander students: 8.1%	Likert-based survey (students): engagement, appropriateness, and perceived efficacy; Qualitative: semistructured interviews with teachers; teacher logbooks postlesson; facilitator observations	Culturally inclusive online or web-based story and lesson content	Assessed both factors influencing engagement and perceived effectiveness of DMHI. Engagement: Illustrations, story, or characters cited in 66.7% of responses as what students enjoyed most Preferences expressed for interactive activities such as the commenting tool A total of 50% of Aboriginal and Torres Strait Islander students and 45.1% non-Indigenous students found the program content relevant to their own lives A total of 53.3% of Aboriginal and Torres Strait Islander students and 63.9% of non-Indigenous students reported the likelihood to use the information and skills taught in the program The facilitators observed that Indigenous students were particularly engaged with cultural elements, such as the importance of older adults and aspects of Aboriginal culture included in stories Non-Indigenous students appreciated cultural education Detailed subgroup analysis was hindered by the small sample size of Indigenous students compared to the overall sample, leading to difficulties in generalizing findings regarding engagement patterns stratified by ethnicity Rural school issues: technological barriers hindered rural school participation in the study Effectiveness and perceived efficacy: A total of 80.5% of students found the content somewhat or very helpful when dealing with peer pressure A total of 78.6% of students found it helpful for stress A total of 85.2% of students found the content helpful for dealing with alcohol and drugs Qualitative data themes: Four key themes regarding acceptability: engagement, appropriateness, content and structure acceptability, and perceived efficacy Four key themes regarding feasibility: implementation fidelity, program length and timing, ease of implementation, and functionality Co-design and cultural relatability work: program developed over 3 years in partnership with an Aboriginal and Torres Strait Islander creative agency, co-designed with 53% Torres Strait Islander and Aboriginal students and 47% non-Indigenous students at 4 schools Extensive stakeholder consultations held included activities, such as photovoice sessions and participatory storytelling such that participants contributed to intervention development Storylines developed with relevant themes and Aboriginal characters for a sense of identification and belonging for Indigenous students
El Morr et al [23], 2020	Canada; randomized controlled trial; 160	Mean age: 22.55 years; White: 20.1%; Black: 14.5%; South Asian: 27.7%; Chinese: 9.4%; other: 28.3%; participants born outside of Canada: 54.7%	PHQ-9; Beck Anxiety Inventory; Perceived Stress Scale; Five Facets Mindfulness Questionnaire short form	Web-based or online CBT platform	Predominantly assessed effectiveness in the context of ethnicity, and some general engagement metrics were assessed. Engagement (general): Video content was found to be the most engaging feature of the intervention, moderate use by participants (average of 6 educational and 6 mindfulness videos watched per week) Videoconferencing had low attendance and declined in attendance over time Forums: completely unused; 54% of students completed at least 50% of the available videos General preference for self-directed learning and practice over commenting tool or social components Effectiveness in young people of different ethnicities (mindfulness): Post-intervention, students under “other” ethnicity had lower mindfulness scores compared to White students (β =−6.56; P=.04) Participants born outside of Canada had lower mindfulness scores (β=−5.89; P=.03) Students whose first language was not English had lower mindfulness scores (β=−5.97; P=.01) Depression and anxiety symptoms: Participants born outside of Canada had higher depression (β=2.96; P=.02) and anxiety (β=4.91; P=.02) scores Cultural relatability and coproduction: Minimal–some focus groups were held with students to assess general student challenges, for example, stress, anxiety, procrastination, and identified from needs assessments No explicit mention of cultural adaptation
Graham et al [24], 2023	United States; Cluster randomized trial; 690	Age as not directly reported; the study cohort comprised college students in the United States.; White: 60.0%; Asian or South Asian: 17.1%; Black or African American: 5.4%; Native Hawaiian or Pacific Islander: 0.1%; American Indian or Alaskan native: 0.4%; multiracial: 7.7%; other races: 6.7%	Weight Concerns Scale; Clinical Impairment Assessment; Perceived Benefits of Thinness Scale; PHQ-9; Patient-reported outcomes; Measurement Information System anxiety short form; Motivation for treatment; Eating Disorder Examination Questionnaire	Digital CBT-guided self-help intervention	Predominantly assessed effectiveness in the context of ethnicity Engagement: not directly measured or assessed Participant retention: 78% in the intervention group and 89% in the control group. Effectiveness in young people of different ethnicities: The moderating effects of race were explained by BMI, which remained significant at follow-up (β=.19; *P*=.03). Cultural relatability and coproduction: not incorporated into design
Gray et al [20], 2024	United States; cross-sectional; 169	Median age: 20 years; White: 53.85%; Asian: 28.99%; Black or African American: 4.14%; Latinx: 8.28%; Native Hawaiian or Pacific Islander: 0.59%; other: 0.59%; missing: 3.55%	Passive data metrics (sleep duration, home time, and screen duration); number of activities completed; number of mindfulness activities completed	Mindfulness-based mental health app	Predominantly assessed engagement in the context of ethnicity. Engagement: Race or ethnicity was not associated with app engagement. Engagement assessed through passive and app-based metrics: estimated time spent asleep, time spent with phone screen on, and time spent at home App-based metrics: completion of mindfulness and nonmindfulness activities and how many days a week activities were completed Regression analysis showed engagement seemed best assessed from estimated sleep duration (P<.03) and screen duration (P<.01), both associated with increased measures of engagement (eg, completion of activities in app) Engagement highly individualized on heat map–k-means clustering showed poor predictive performance for passive metrics–engagement patterns not easily grouped into generalizable clusters: All participants: adjusted rand index=0.008; silhouette score=0.41 Participants who completed mindfulness activities: adjusted rand index=−0.002; silhouette score=0.39 Cultural relatability and coproduction: Not mentioned in the development of app or paper

^a^GAD-7: Generalized Anxiety Disorder-7 Item Scale.

^b^PHQ: Patient Health Questionnaire.

^c^MYAS: Mentor-Youth Alliance Scale.

^d^CBT: cognitive behavioral therapy.

^e^DMHI: digital mental health intervention.

^f^PANAS: Positive and Negative Affect Scale.

^g^K10: Kessler Psychological Distress Scale.

^h^WOB: Ways of Being.

### Demographics

The age of most study participants ranged from 12 to 25 years. Two studies were conducted in university settings, resulting in some data collected from persons aged >25 years. In the study by Ahuvia et al [19], 95% of participants were aged between 18 and 25 years, and in the study by El Morr et al [23], the study population had a mean age of 22.55 years. Six studies recruited >100 participants, with 1 US-based study recruiting >1000 participants.

Ethnic demographics varied substantially between studies. Only 2 studies had populations that were predominantly racial and ethnic minorities [21,23], while the others consisted mostly of White participants. In 6 studies, the most common minority ethnic group represented were those who identified as “Asian.” Overall, sex demographics in these studies skewed toward predominantly female participants, with only the study by Routledge et al [21] having an equal sex ratio and 1 study assessing an eating disorder intervention with only female participants [24]**.**

There were a variety of methodologies adopted by the included studies; 2 studies used a mixed methods approach, 2 were cross-sectional studies, 2 were randomized controlled trials, and 1 was a cluster randomized trial [24]. This resulted in outcomes being reported in a mixture of qualitative and quantitative formats, with a variety of psychological measures and research tools used to explore these outcomes.

Not all included studies assessed both the engagement and effectiveness of the DMHI. Two studies solely explored engagement factors [19,20], 3 studies solely explored effectiveness outcomes following DMHI use [22-24], and 2 studies explored both engagement factors and effectiveness outcomes [21,25]. Ahuvia et al [19] focused solely on assessing the factors influencing initial engagement with DMHIs, while Syed Sheriff et al [22], El Morr et al [23], and Graham et al [24] explored the effectiveness of DMHIs in the context of psychological outcomes. In addition, Gray et al [20] assessed engagement with a mindfulness-based app as the number of activities completed by users.

### Digital Interventions and Initial Overview

All the digital interventions studied required internet access and were web based. Four studies were web-based adaptations of cognitive behavioral therapy (CBT; eg, Graham et al [24]), and 75% (3/4) of these assessed the effectiveness of DMHIs on improving psychological symptomatology and only 25% (1/4) of these CBT-based interventions explored engagement factors. Of these, Graham et al [24] found that any differences between race and ethnicity on the effectiveness of a digital eating disorders intervention became nonsignificant when BMI was accounted for. El Morr et al [23] found that those students who fell into the “other—non-White” category had significantly lower mindfulness scores (β=−6.56; *P*=.04) compared to White students when accessing their web-based CBT platform. Giovanelli et al [25] provided the CBT program via a near-peer mentorship program, where young people were mentored by individuals from similar sociocultural backgrounds. Significant proportions of participants reported the mentorship aspect of the intervention to be beneficial. Two other included studies involved the use of culturally relevant stories for promoting psychological wellness; one explored both engagement and effectiveness metrics, while the other solely focused on the effectiveness of the DMHI for young people of different ethnicities. Routledge et al [21] used web-based culturally relevant storytelling to deliver an alcohol and drug prevention program to an Australian community and found great success in both engagement and effectiveness, assessed by researcher questionnaires distributed to students. Syed Sheriff et al [22] delivered a web-based cultural experience to promote youth well-being through the exploration of history and the arts, and their study found significant differences in negative affect for ethnic minority youths after accessing their DMHI (−0.45 treatment effect on negative affect after accessing the DMHI; 95% CI −0.6 to −0.2).

Culturally relevant content and low-cost options were associated with higher engagement (3/7, 43%) and improved psychological outcomes (3/7, 43%). Specifically, interventions that incorporated elements such as near-peer mentorship and cultural tailoring demonstrated significant improvements in effectiveness outcomes, such as Generalized Anxiety Disorder-7 Item Scale (GAD-7), Patient Health Questionnaire (PHQ), and negative affect scoring (3/7, 43%). Language barriers, cost, and lack of cultural relatability were identified as significant factors that could hinder both the engagement with (2/7, 29%) and effectiveness of DMHI for nonnative English speakers and those from minority backgrounds [23].

### Outcome Measures and Research Tools

Assessment of engagement between studies that explored engagement factors was heterogenous (4/6, 57%); some studies used researcher-designed feedback questionnaires (2/4, 50%), while 1 (25%) used the net promoter score measure, and another study quantified the number of activities completed within an app (n=1, 25%). Most studies (5/7, 71%) that assessed effectiveness used validated measures, such as the GAD-7, PHQ, and Eating Disorder Examination (4/5, 80%). There was variation between studies in the number of quantitative measures used, with the mean number of outcome measures and research tools used across 6 of the studies being 3.8 (SD 2.1). Other measures used tended to be specific to the digital intervention being explored; for example, the internet-based mentorship program developed in the study by Giovanelli et al [25] used the mentor-youth alliance scale to assess the strength of relationships between the youth and the mentor, while El Morr et al [23] used the Five Facets mindfulness questionnaire to assess the efficacy of their web-based CBT platform.

In terms of qualitative measures, Giovanelli et al [25] conducted semistructured interviews with young people alongside quantitative measures. Questions focused on their experience using the DMHI, Appa Health, including areas where it could expand to better fit the needs of young people, and experience navigating and using the digital content, including videos. Routledge et al [21] used semistructured interviews with teachers to explore program effectiveness on students and used teacher logbooks and facilitator observation forms to assess program fidelity.

There were further differences between studies insofar as which reported outcomes achieved significance because of the range of specific tools deployed for each digital intervention. Despite several studies measuring PHQ and anxiety outcomes, only Giovanelli et al [25] reported significant reductions in PHQ and GAD-7 scoring from their program. Others [22,23] reported significant differences in areas such as mindfulness and negative affect scoring for ethnic minority youth, which was the focus of the specific digital intervention being deployed.

### Results of Individual Studies

The study by Ahuvia et al [19] explored potential factors impacting initial interest and engagement with at-cost and free forms of teletherapy or self-help. PHQ-9 and GAD-7 measures were used to assess whether depressive or anxiety symptoms correlated with interest in digital mental health support, and a web-based quantitative questionnaire was disseminated with a binary yes or no scale to indicate participant interest in the DMHI. Logistic regression was used for analysis. White, non-Hispanic students were used as a reference category for the generation of odds ratios. The sample size was the largest of any of the studies included (n=1224), with most participants (60%) originating from ethnic minority backgrounds. All minority ethnic groups studied (Black, Asian, Hispanic, and other populations with racial and ethnic minorities) showed higher interest in free forms of digital mental health support compared to the reference category, though almost none of these comparisons achieved statistical significance. Solely Asian students showed statistically significantly lower interest in at-cost teletherapies compared to reference (odds ratio 0.45; *P*<.001). The effectiveness of these interventions was not explored by this study, and no cultural adaptations or relatability procedures were conducted for any DMHIs.

Giovanelli et al [25] developed a smartphone app with CBT video content and a near-peer mentorship program, where young people were matched to mentors of a similar sociocultural background. Due to this being a pilot usability study, it explored the smallest sample size out of all the studies included in the review (n=14), and it simultaneously explored the experiences of lesbian, gay, bisexual, transgender, queer, and similar minority (LGBTQ+) youth mentorship as well as ethnic minority youth mentorship. The ethnic demographics for this study were 64% White. Subgroup analysis of engagement was impeded by limited sample size, and only qualitative responses were given, which suggested that choice of mentor from a similar minority group, for example, ethnicity, was positively received. Participants highlighted app accessibility and ease of use (eg, flexibility of scheduling mentoring sessions and short-form video content) as positive experiences. All participants completed the 12-week program and surveys at all time points, showcasing strong program retention and sustained engagement. A median of 107 minutes over a 12-week period was spent in video calls between mentors and mentees, and 100% of participants expressed positive responses to the short-form (30-90 seconds) CBT video content. Four validated tools were used to explore the effectiveness outcomes related to psychological sequelae and mentorship experiences. There were significant reductions in median PHQ-8 and GAD-7 scores for participants, and 100% of participants stated that the mentoring aspect was particularly beneficial. Semistructured interviews with participants indicated that combining mentoring with CBT strategies had the potential to positively affect youth mental health.

This intervention was developed using input from youth and clinical advisory boards, whereby youth advisors provided feedback on the relatability and engagement of the app content and mental health professionals ensured clinical accuracy of content. Mentors were selected for relatable lived experiences from a diverse range of backgrounds; 50% identified as Hispanic, Latinx, or other and 30% as LGBTQ+. Video content was designed in a short-form format to promote youth engagement, and participant feedback loops, customization, and choice of mentor enabled personalization of the program experience for young people of different ethnicities. This was well received by young people from diverse ethnic backgrounds, who qualitatively reported that having a mentor of similar heritage was important to them.

A study by Syed Sheriff et al [22] compared a culturally adapted web-based experience of the Ashmolean Museum to the standard website of the museum and how this would impact the mental health of young people as a health promotional activity. This was titled “Ways of Being,” and was coproduced over a 3-month period using an iterative process; key stakeholders involved in the development of ways of being included young people aged 16 to 24 years, museum curators, youth engagement officers, and education officers. Stakeholder workshops were held to gather diverse viewpoints, with 12 young people selected to represent diverse ethnic backgrounds.

There was no subgroup analysis conducted related to engagement due to data security. General engagement metrics showed that 75% of participants completed all assessments regarding content at the 6-week follow-up, suggesting a high retention rate. However, content exhaustion was highlighted at this point, whereby participants felt they had exhausted all available content before the end of the intervention period.

The effectiveness of the DMHI for young people was assessed through feedback questionnaires and psychological scale measures, such as the Kessler Psychological Distress Scale. There were significant differences in negative affect scoring for ethnic minority groups when accessing the culturally adapted experience compared to the standard website (−0.45 treatment effect on negative affect after accessing intervention; 95% CI –0.6 to –0.2), as well as improvements in mean psychological distress scores across the population when accessing the cultural experience. However, it must be noted that the sample was a majority White population (78%), which limits the generalizability of these findings.

Routledge et al [21] disseminated a culturally adapted web-based series of lessons related to drug and alcohol prevention to a cohort of youth aged 12 to 14-years in Australia over 6 weeks, with coproduction of the curriculum involving students and staff from Aboriginal and Islander communities as well as non-Indigenous individuals. The program was developed over 3 years alongside Aboriginal and Torres Strait Islander creative agencies and co-designed with 53% Torres Strait Islander and Aboriginal students and 47% non-Indigenous students at 4 schools (2 urban and 2 rural). Stakeholder consultations were held with Indigenous representation, which informed the program content, structure, and character design. Storylines were developed with relevant themes and Indigenous characters for a sense of belonging for Indigenous students, and participatory storytelling enabled participants to contribute to intervention development.

Detailed demographics for the study were not outlined, beyond the cohort consisting of 8.1% Aboriginal and Torres Strait Islander students. Both engagement and effectiveness outcomes in students were predominantly assessed via qualitative surveys, and facilitator observation informed further assessment of engagement. Students found the culturally adapted stories, characters, and illustrations to be the most engaging and enjoyable element of the program (66.7%). Facilitator observations indicated that Indigenous students were engaged with cultural elements and aspects of Indigenous culture included in storytelling, while non-Indigenous students appreciated the cultural education. In total, 50% of Indigenous and 45.1% of non-Indigenous students found the content relevant to their lives. Detailed subgroup analysis was hindered by the relatively small sample size of Indigenous students compared to the overall sample size, which led to difficulties generalizing the findings regarding engagement.

Students rated the perceived effectiveness of the DMHI based on how helpful the content was across key content areas: 80.5% of students found the content helpful when dealing with peer pressure, 78.6% of students found the content helpful for dealing with stress, and 85.2% of students found the content helpful for dealing with alcohol and drugs.

The study by El Morr et al [23] explored an 8-week program delivering video CBT modules and anonymized web-based forums to a cohort of 160 undergraduate students from Canada. Engagement was reported in general terms, with no subgroup analysis conducted by demographic factors such as ethnicity or race. Video content was found to be the most engaging feature of the intervention, with moderate use by participants (an average of 6 educational and 6 mindfulness videos watched per week). In total, 54% of students completed at least 50% of the available videos, and there was a general preference for self-directed learning and practice over the commenting tool or social components. Four psychological measures were deployed to assess effectiveness outcomes related to perceived stress, mindfulness, and depression and anxiety symptoms. Demographically, the cohort predominantly consisted of participants born outside of Canada and who identified as racial and ethnic minorities. It was found that those students who identified as “other, non-White” ethnicities had significantly lower mindfulness scores compared to White students after using the program (β=−5.89; *P*=.03). Those participants whose first language was not English had worse mindfulness (β=−5.97; *P*=.01) scores after accessing the intervention. There were no explicit mentions of cultural adaptation; however, some focus groups were held to assess the general challenges faced by the student population (eg, stress, anxiety, and procrastination) and mental health needs identified from needs assessments, which then informed the content of the app.

A study by Graham et al [24] examined the moderators and mediators of an effective digital CBT-guided self-help intervention for college women with an eating disorder, the Student Bodies–Eating Disorders intervention. Findings indicated that participants who identified as Black experienced less improvement in eating disorder psychopathology compared to their White counterparts (β=.72; *P*=.06). Conversely, Asian participants showed slightly greater improvement relative to White participants (β=−.42; *P*=.07). However, these moderating effects of identifying as Black or Asian compared to White were attenuated when adjusted for BMI. This study also possessed a majority White population (60%). Engagement factors were not directly assessed in this study, though participant retention rates were given, with 78% participant retention in the intervention group and 89% in the control group. Cultural relatability, focus groups, or elements of coproduction were not mentioned in the design of the study or the DMHI.

A study by Gray et al [20] examined the patterns of mindfulness-based app engagement among college students across 2 iterations of a 4-week study. Engagement was assessed through passive and app-based metrics, where passive metrics included estimated time spent asleep, screen time (time spent with phone screen on), and time spent at real-world home location. No significant associations were found between race or ethnicity and mindfulness app engagement. Measures of engagement with the app included the total number of mindfulness activities completed that week, the proportion of days that week in which mindfulness activities were completed, and the proportion of days that week in which any activity (including nonmindfulness activities) was completed. Nonmindfulness activities included accessing psychoeducation material in the app or CBT-related exercises completed in the app. Overall, the results indicated that race and ethnicity do not significantly influence engagement in this context. However, k-means clustering of the datasets suggested that the factors influencing engagement may be highly individualized; there was poor predictive performance for passive metrics (all participants: adjusted rand index=0.008; silhouette score=0.41 and participants completing mindfulness activities: adjusted rand index=−0.002; silhouette score=0.39). There were no mentions of cultural adaptations, co-design, or stakeholder consultations in the development of the study or the app.

## Discussion

### Principal Findings

To the best of our knowledge, this is the first systematic review exploring the engagement and effectiveness of DMHIs among young people of different ethnicities. Seven studies met the inclusion criteria. All included studies were conducted in high-income countries. Culturally adapted, low-cost interventions demonstrated greater interest, engagement, and effectiveness. However, the limited number of studies and small sample sizes hindered comprehensive comparisons. Studies that had addressed this question were largely cross-sectional or small-scale evaluations, meaning that comparison with White populations or subanalyses by ethnicity could not be performed. Studies varied in their outcomes, with some (2/7, 29%) primarily focusing on engagement with DMHIs, others predominantly addressing effectiveness (3/7, 43%), and 2 (29%) exploring both. Some studies (2/7, 29%) provided general data on engagement alongside their analyses of effectiveness. Strategies such as coproduction, mentorship, and personalized support show promise in enhancing cultural relevance, thus providing more engaging and effective DMHIs.

### Cultural Relatability and Low-Cost Interventions

Results indicated that cultural relatability (including culturally relevant content and stories) appeared to play a role in promoting both user engagement and effectiveness, as particularly evidenced in the studies by Syed Sheriff et al [22] and Routledge et al [21]. Low-cost interventions also appeared to garner increased interest from young people of different ethnicities, though there were no studies directly exploring the treatment effect of low-cost interventions. We also found that those whose native language was not that of the disseminated digital intervention received less benefit compared to the standard population, which suggests the importance of linguistic adaptation in the development of globally capable DMHIs.

Current literature shows conflict regarding the importance of cultural relatability of digital interventions and whether this offers quantifiable benefit to engagement and experiences of minoritized youth when accessing DMHIs [26]. In our review, cultural relatability preliminarily appeared to play a role in improved engagement and outcomes following use of a DMHI. Routledge et al [21] highlighted the potential for coproduction as a viable strategy for improving relatability. Coproduction has been found to be an effective tool for the development of mental health resources in other areas and, therefore, may be of future benefit to improve user engagement as interventions continue to evolve [27]. Several frameworks for community involvement in the development of digital mental health resources have been proposed, such as combining motivational interviewing with cultural assessment [28,29]. Despite this, coproduction has not yet been widely used in the context of DMHIs [30]. We suggest this should be considered when developing these interventions with young people of different ethnicities in mind.

The significant interest in free forms of digital mental health care aligns with the suggestion of socioeconomic factors acting as barriers to health care access for minoritized individuals, highlighting that this issue is not restricted to the adult population [19,26,31]. This warrants further exploration through both dissemination of low-cost digital interventions in this group as well as qualitative research into the factors influencing initial uptake of digital interventions in young people of different ethnicities, as this may uncover insights into how these barriers arise and how to effectively remediate them. For student populations, university campuses have been suggested as effective areas to disseminate mental health care and mitigate disparities in access for young people of different ethnicities [11].

Our review additionally explores the experiences of school-aged children and suggests that school-based and educational interventions may be effective in addressing disparities in DMHI adoption for this age group. However, when an intervention is not adapted linguistically or socioculturally, it may be less effective for individuals who are not native English speakers or who belong to minority ethnocultural groups. Further work should be conducted to explore the efficacy of the school setting for addressing disparities in accessing mental health resources for ethnic minority youth. The study by Routledge et al [21] offers a promising insight into how digital classrooms can be used for mental health support.

### Role of Digital Interventions

Some concerns may be raised as to whether these DMHIs would act as replacements for accessing necessary, physician-led mental health care [32]. In our review, the most successful digital interventions studied predominantly acted as health promotion or preventative exercises, presented through a culturally accessible lens for minoritized young people. Moreover, young people often attribute poor engagement with digital interventions to the lack of personalized psychological support, emphasizing the need for a human component in the mental health care process [26,33]. This is further supported by our review, where the study by Giovanelli et al [25] found significant benefits from their mobile appl being supplemented by near-peer mentorship for the participants.

Despite health inequities being a global health priority according to both the WHO and National Health Service (NHS) [9,34], very few studies directly compared the responses of young people of different ethnicities to those of White groups when accessing DMHIs. There is a need to explore whether ethnicity acts as a predictor of treatment response to these interventions, as if these interventions are not equitable across ethnicities, they may exacerbate existing health disparities [34]. Moreover, detailed insight into ethnic differences in the treatment response to these interventions will further inform health policy and practice while enhancing engagement.

Global pressures on health care systems mean that strategic deployment of supplementary technologies may serve to alleviate some of the burden on services at present [35]. Moreover, the anonymity and ease of access of technologies, such as free mobile apps and websites, may provide methods to remediate both socioeconomic and stigma-related barriers for ethnic minority youth when accessing traditional health care, though evidence in this area continues to be sparse [36,37].

### Limitations of the Review

This review provides an initial insight into an area of need identified by the WHO and is novel in its specific exploration of experiences of young people of different ethnicities. A significant limitation of the included studies was that all the included studies were conducted in high-income countries and possessed limited representation of minority youth participants. This resulted in limited external validity, underpowered subgroup analyses, and limited generalizability, as despite some large sample sizes, imbalances in ethnic representation may result in skewed findings regarding the factors influencing engagement and effectiveness of DMHIs in ethnic minority youth. Future studies may address this by oversampling individuals from underrepresented groups, building partnerships with cultural or religious community leaders to facilitate coproduction of resources, producing low or no cost DMHIs, and creating multilingual tailored outreach documents and DMHIs, as linguistic and financial barriers played a key role in both engagement and effectiveness outcomes across multiple studies in our review.

Furthermore, there appear to be very few qualitative analyses conducted on the factors influencing the initial uptake of DMHIs among young people of different ethnicities. Moreover, none of the studies included explored the impact of DMHI on children of different ethnicities aged <12 years. Early identification and intervention for mental health challenges in this age group could prevent deterioration and improve long-term outcomes. Future research should explore how DMHIs can be tailored to meet the needs of younger children, focusing on factors such as age-appropriate content, parental involvement, and usability. There was also significant heterogeneity between studies in the assessment of user engagement and effectiveness, therefore limiting comparability between the studies. Some studies opted for researcher-designed questionnaires, while others favored standardized and commonly accepted measures such as GAD-7 to measure effectiveness.

A notable limitation in the existing literature is the lack of a uniform definition of what constitutes a DMHI. This ambiguity complicates the process of developing comprehensive search terms and strategies that could target all potential studies relating to DMHI. This underscores the need for a consensus among researchers as to what constitutes a DMHI. While we have adopted our working definition of a DMHI to facilitate a systematic search strategy, this definition may evolve as the use and development of DMHIs expands and the potential role of artificial intelligence (AI) in digital mental health crystallizes. We did not limit the methodologies of the included studies, and therefore, our review provides a broad overview of the subject area as opposed to a focused exploration of either qualitative or quantitative research. There were also insufficient studies with the same outcome to perform a meta-analysis.

### Future Implications for Practice and Research

#### Developing a Standardized DMHI Definition

Given the rapid advancements in technology, including AI and robotics, it is essential to clearly define which technologies fall within the scope of DMHIs. Establishing this clarity will provide researchers with a consistent foundation for analysis. Developing a clear definition may assist in the development of culturally adapted DMHI and improvement of engagement in young people of different ethnicities [30]. We suggest that DMHIs should be defined as we have done for our search strategy within this review: digitally delivered products designed for either preventative, diagnostic, therapeutic, or psychological benefit of an individual’s mental health. This definition should be inclusive of AI as a digital technology, as this is a newly evolving field that may play a key role in the development of new DMHIs.

#### Developing Key Indicators for Engagement and Effectiveness of DMHIs

Lack of standardized measures of engagement hindered significant comparisons being drawn between studies in this review. The most effective methods for assessing engagement appear to come from mixed methods approaches, incorporating both digital phenotyping and methods such as focus groups, interviews, or researcher-designed subjective questionnaires. It would be proposed that the following key indicators be considered when assessing engagement with DMHIs, on the basis of the different tools used by the studies included in this review: “behavioral engagement,” “emotional engagement,” “cognitive engagement,” “audio-visual engagement,” and “social engagement.” Behavioral engagement refers to the user retention and dropout rates, which could be assessed via digital phenotyping (eg, application behaviors, passive data, and activities completed). Emotional engagement refers to the enjoyment, relatability, and authenticity of the DMHI, with authenticity being particularly relevant in the context of culturally relatable experiences being included in DMHIs; these could be assessed via researcher-designed questionnaires, interviews, or focus groups conducted during and after intervention use. Cognitive engagement refers to the attention time held of the DMHI users, which could be assessed via digital phenotyping (eg, time taken to complete an activity and time spent on DMHI). Audio-visual engagement refers to how users experience the audio-visual design—whether the design is comforting or appealing and supporting their enjoyment of the DMHI, as this appeared to play a role in several of the studies included in this review; this could be assessed via questionnaires or interviewing. Social engagement refers to those DMHIs where social elements are involved (eg, mentorship or peer interaction), where users may be queried on the experiences of their interactions with others, comfort levels, and the usefulness of social features within the DMHI; this may be assessed through researcher-designed questionnaires or participant interviewing.

Heterogeneity in the assessment of effectiveness was to be expected due to the differences in intended uses and purposes of DMHIs. To facilitate streamlined assessments of DMHI effectiveness, we suggest that the key areas of effectiveness of DMHIs include the following: “psychological symptom improvement,” “knowledge or skills gained,” “integration or behavioral modifications,” “perceived effectiveness and satisfaction,” and “cultural and social relatability and community.” Psychological measures were used across most studies within this review as validated tools to explore the effectiveness of DMHIs, and we suggest that these continue to be used before, during, and after intervention implementation for monitoring the effectiveness of the DMHI on symptomatic improvement. “Knowledge or skills gained” relates to those DMHIs where user education is a priority and can be assessed via interviewing and questionnaires to assess participant knowledge before, during, and after intervention implementation. “Integration or behavioral modifications” incorporate whether the DMHI has been adopted and integrated into the participant’s life and if any changes have been observed because of this; this facet can be explored via interviewing, focus groups, or researcher-designed questionnaires. “Perceived effectiveness and satisfaction” highlights whether the individual found the DMHI to be an impactful experience on their life and could be assessed via subjective surveys and interviewing; the individual’s perceived experience of a DMHI may differ from the objective psychological measures. “Cultural and social relatability and community” explores whether any cultural elements within the DMHI felt relatable or produced a sense of connection or community or whether cultural elements improved the understanding of other cultures; these could be explored via interviewing and focus groups.

#### Increasing Cultural Relatability

Within our review, those DMHIs that incorporated a significant number of stakeholder consultations or workshops from early development appeared to have high levels of engagement, enjoyment, and effectiveness for young people of different ethnicities. It is suggested that stakeholders from a diverse array of ethnic backgrounds be incorporated into the audio-visual design, methods of delivering content, and development of content for future DMHIs. Inclusion of culturally relatable individuals also appeared to play a role across multiple studies, whether this was a fictional character, historical figure, or near-peer mentor. Coproduction with community organizations and leaders, cultural arts and heritage groups, and stakeholders appears to play a significant role in both engagement and effectiveness. Actively involving young people in the process of designing and developing DMHIs may also play a key role in understanding and incorporating these individual factors into future DMHIs. Moreover, qualitative investigation into these individual factors that motivate engagement should be explored in future research into DMHIs in young people of different ethnicities. Short-form content appeared to be preferred by all youth in the DMHIs that implemented multimedia such as videos and would be suggested as the preferred form of media for future DMHIs.

#### Further Work

Furthermore, qualitative analyses exploring both the motivating factors for initial use and reuptake of DMHIs in young people of different ethnicities can be conducted, as there were significant variations in both the demographic makeup and number of participants between studies. Broadly, analyses need to be conducted with larger sample sizes and more diverse populations to ensure statistical power and validity of findings. In addition, studies should explicitly aim to examine differences by ethnicity within their design, with sufficiently large subgroups and analysis plans to support this, rather than relying on post hoc reporting by ethnicity. Qualitative studies would also enable researchers to conduct in-depth explorations of the importance of cultural relatability in the context of DMHIs.

Future studies may need to consider additional factors that contribute to the differential uptake of DMHIs across ethnic groups. These could include digital exclusion and literacy, limited access to private space or personal devices, restrictions on device use, unreliable internet access, varying levels of trust in digital mental health resources, and data security concerns. In addition, health inequalities related to sex and comorbid neurodevelopmental disorders may play a role.

While some studies highlighted the importance of culturally adapted interventions, there remains a gap in understanding the specific elements that make these adaptations successful. Future research should focus on identifying and testing specific cultural adaptation strategies, such as incorporating culturally relevant content, use of language interpreters instead of direct translation, and community coproduction in the design and implementation of DMHIs. Pilot studies and randomized controlled trials that systematically compare adapted versus nonadapted interventions can provide empirical evidence on the effectiveness of these strategies.

Finally, both our review and the WHO guidelines [9] has highlighted a paucity of research into the relationship between children of different ethnicities aged <12 years and DMHIs; future research should uncover whether there is a potential for DMHIs to create a significant benefit within this age group and any potential risks associated with the use of DMHI in this cohort.

### Conclusions

In conclusion, this review offers insight into the potential of DMHI as a supportive tool for the mental health of young people of different ethnicities. The studies included in this review collectively indicate the potential of DMHI in young people of different ethnicities, particularly when culturally adapted. The greatest benefits appear to occur when DMHIs are coproduced to encourage relatability and engagement with young people and when children are able to interact with relatable near-peer individuals to guide them through mental health experiences. Several gaps in the literature have been identified by our review, particularly that the field would benefit from a consensus definition of what constitutes a DMHI. Furthermore, there is still a paucity of research within this area, and therefore, more extensive qualitative reporting of the experiences of young people of different ethnicities accessing DMHIs is needed, alongside studies powered to identify differences in treatment response by ethnicity.

## Appendix

Multimedia Appendix 1 Search strategy for this paper.

Multimedia Appendix 2 PRISMA checklist.

## Abbreviations

AI: artificial intelligence
CBT: cognitive behavioral therapy
DMHI: digital mental health intervention
GAD-7: Generalized Anxiety Disorder-7 Item Scale
LGBTQ+: lesbian, gay, bisexual, transgender, queer, and similar minority
MMAT: Mixed Methods Appraisal Tool
PHQ: Patient Health Questionnaire
PRISMA: Preferred Reporting Items for Systematic Reviews and Meta-Analyses
WHO: World Health Organization

## Data Availability

The search strategy is available in Multimedia Appendix 1.
