# Supplementary material for: Exploring Engagement With and Effectiveness of Digital Mental Health Interventions in Young People of Different Ethnicities: Systematic Review
Source: J Med Internet Res. 2025 Apr 7;27:e68544. doi: 10.2196/68544 (PMC12012393; doi:10.2196/68544)
Supplement: Multimedia Appendix 1 [file jmir_v27i1e68544_app1.docx]

**Appendix 1 – Search Strategies & Terms**

**PsycINFO Search Strategy**

**Digital Mental Health**

1. exp Mental Health/ or exp Mental Disorders/

2. digital mental health.mp. [mp=title, abstract, heading word, table of contents, key concepts, original title, tests & measures, mesh word]

3. exp Therapy, Computer-Assisted/

4. exp distance counseling/ or exp mental health teletherapy/

5. exp Internet-Based Intervention/

6. e-mental health.mp.

7. digital intervention.mp.

8. online therap*.mp.

9. etherap*.mp. [mp=title, abstract, heading word, table of contents, key concepts, original title, tests & measures, mesh word]

10. web-based intervention*.mp. [mp=title, abstract, heading word, table of contents, key concepts, original title, tests & measures, mesh word]

11. electronic health service*.mp. [mp=title, abstract, heading word, table of contents, key concepts, original title, tests & measures, mesh word]

12. mhealth.mp. [mp=title, abstract, heading word, table of contents, key concepts, original title, tests & measures, mesh word]

13. mobile app*.mp. [mp=title, abstract, heading word, table of contents, key concepts, original title, tests & measures, mesh word]

14. phone app*.mp. [mp=title, abstract, heading word, table of contents, key concepts, original title, tests & measures, mesh word]

**Young People**

15. Adolescent/ or young people.mp. or Child/

16. young pe*.mp. [mp=title, abstract, heading word, table of contents, key concepts, original title, tests & measures, mesh word]

17. adole*.mp. [mp=title, abstract, heading word, table of contents, key concepts, original title, tests & measures, mesh word]

18. child*.mp. [mp=title, abstract, heading word, table of contents, key concepts, original title, tests & measures, mesh word]

19. student.mp. or Students/

**Mental Health further identifiers**

20. mental health.mp. [mp=title, abstract, heading word, table of contents, key concepts, original title, tests & measures, mesh word]

21. mental disorder*.mp. [mp=title, abstract, heading word, table of contents, key concepts, original title, tests & measures, mesh word]

22. psych*.mp. [mp=title, abstract, heading word, table of contents, key concepts, original title, tests & measures, mesh word]

**Engagement and Experience**

23. Attitude/ or attitude.mp.

24. response.mp.

25. experienc*.mp.

26. engagement.mp.

**Ethnicity**

27. ethnic*.mp. or exp "Ethnic and Racial Minorities"/ or exp Ethnicity/

**Combination of terms for final search**

28. 23 or 24 or 25 or 26

29. 1 or 20 or 21 or 22

30. 15 or 16 or 17 or 18 or 19

31. 2 or 3 or 4 or 5 or 6 or 7 or 8 or 9 or 10 or 11 or 12 or 13 or 14

32. 27 and 28 and 29 and 30 and 31

**MEDLINE Search Strategy**

**Digital Mental Health**

1. exp Mental Health/ or exp Mental Disorders/

2. digital mental health.mp. [mp=title, book title, abstract, original title, name of substance word, subject heading word, floating sub-heading word, keyword heading word, organism supplementary concept word, protocol supplementary concept word, rare disease supplementary concept word, unique identifier, synonyms, population supplementary concept word, anatomy supplementary concept word]

3. exp Therapy, Computer-Assisted/

4. exp distance counseling/ or exp mental health teletherapy/

5. exp Internet-Based Intervention/

6. e-mental health.mp.

7. digital intervention.mp.

8. online therap*.mp.

9. etherap*.mp. [mp=title, book title, abstract, original title, name of substance word, subject heading word, floating sub-heading word, keyword heading word, organism supplementary concept word, protocol supplementary concept word, rare disease supplementary concept word, unique identifier, synonyms, population supplementary concept word, anatomy supplementary concept word]

10. web-based intervention*.mp. [mp=title, book title, abstract, original title, name of substance word, subject heading word, floating sub-heading word, keyword heading word, organism supplementary concept word, protocol supplementary concept word, rare disease supplementary concept word, unique identifier, synonyms, population supplementary concept word, anatomy supplementary concept word]

11. electronic health service*.mp. [mp=title, book title, abstract, original title, name of substance word, subject heading word, floating sub-heading word, keyword heading word, organism supplementary concept word, protocol supplementary concept word, rare disease supplementary concept word, unique identifier, synonyms, population supplementary concept word, anatomy supplementary concept word]

12. mhealth.mp. [mp=title, book title, abstract, original title, name of substance word, subject heading word, floating sub-heading word, keyword heading word, organism supplementary concept word, protocol supplementary concept word, rare disease supplementary concept word, unique identifier, synonyms, population supplementary concept word, anatomy supplementary concept word]

13. mobile app*.mp. [mp=title, book title, abstract, original title, name of substance word, subject heading word, floating sub-heading word, keyword heading word, organism supplementary concept word, protocol supplementary concept word, rare disease supplementary concept word, unique identifier, synonyms, population supplementary concept word, anatomy supplementary concept word]

14. phone app*.mp. [mp=title, book title, abstract, original title, name of substance word, subject heading word, floating sub-heading word, keyword heading word, organism supplementary concept word, protocol supplementary concept word, rare disease supplementary concept word, unique identifier, synonyms, population supplementary concept word, anatomy supplementary concept word]

**Young People**

15. Adolescent/ or young people.mp. or Child/

16. young pe*.mp. [mp=title, book title, abstract, original title, name of substance word, subject heading word, floating sub-heading word, keyword heading word, organism supplementary concept word, protocol supplementary concept word, rare disease supplementary concept word, unique identifier, synonyms, population supplementary concept word, anatomy supplementary concept word]

17. adole*.mp. [mp=title, book title, abstract, original title, name of substance word, subject heading word, floating sub-heading word, keyword heading word, organism supplementary concept word, protocol supplementary concept word, rare disease supplementary concept word, unique identifier, synonyms, population supplementary concept word, anatomy supplementary concept word]

18. child*.mp. [mp=title, book title, abstract, original title, name of substance word, subject heading word, floating sub-heading word, keyword heading word, organism supplementary concept word, protocol supplementary concept word, rare disease supplementary concept word, unique identifier, synonyms, population supplementary concept word, anatomy supplementary concept word]

19. student.mp. or Students/

**Further Mental Health Identifiers**

20. mental health.mp. [mp=title, book title, abstract, original title, name of substance word, subject heading word, floating sub-heading word, keyword heading word, organism supplementary concept word, protocol supplementary concept word, rare disease supplementary concept word, unique identifier, synonyms, population supplementary concept word, anatomy supplementary concept word]

21. mental disorder*.mp. [mp=title, book title, abstract, original title, name of substance word, subject heading word, floating sub-heading word, keyword heading word, organism supplementary concept word, protocol supplementary concept word, rare disease supplementary concept word, unique identifier, synonyms, population supplementary concept word, anatomy supplementary concept word]

22. psych*.mp. [mp=title, book title, abstract, original title, name of substance word, subject heading word, floating sub-heading word, keyword heading word, organism supplementary concept word, protocol supplementary concept word, rare disease supplementary concept word, unique identifier, synonyms, population supplementary concept word, anatomy supplementary concept word]

**Engagement and Effectiveness**

23. Attitude/ or attitude.mp.

24. response.mp.

25. experienc*.mp.

26. engagement.mp.

**Ethnicity**

27. ethnic*.mp. or exp "Ethnic and Racial Minorities"/ or exp Ethnicity/

**Term combination**

28. 23 or 24 or 25 or 26

29. 1 or 20 or 21 or 22

30. 15 or 16 or 17 or 18 or 19

31. 2 or 3 or 4 or 5 or 6 or 7 or 8 or 9 or 10 or 11 or 12 or 13 or 14

32. 27 and 28 and 29 and 30 and 31

**EMBASE Search Strategy**

**Digital Mental Health**

1. exp Mental Health/ or exp Mental Disorders/

2. digital mental health.mp. [mp=title, abstract, heading word, drug trade name, original title, device manufacturer, drug manufacturer, device trade name, keyword heading word, floating subheading word, candidate term word]

3. exp Therapy, Computer-Assisted/

4. exp distance counseling/ or exp mental health teletherapy/

5. exp Internet-Based Intervention/

6. e-mental health.mp.

7. digital intervention.mp.

8. online therap*.mp.

9. etherap*.mp. [mp=title, abstract, heading word, drug trade name, original title, device manufacturer, drug manufacturer, device trade name, keyword heading word, floating subheading word, candidate term word]

10. web-based intervention*.mp. [mp=title, abstract, heading word, drug trade name, original title, device manufacturer, drug manufacturer, device trade name, keyword heading word, floating subheading word, candidate term word]

11. electronic health service*.mp. [mp=title, abstract, heading word, drug trade name, original title, device manufacturer, drug manufacturer, device trade name, keyword heading word, floating subheading word, candidate term word]

12. mhealth.mp. [mp=title, abstract, heading word, drug trade name, original title, device manufacturer, drug manufacturer, device trade name, keyword heading word, floating subheading word, candidate term word]

13. mobile app*.mp. [mp=title, abstract, heading word, drug trade name, original title, device manufacturer, drug manufacturer, device trade name, keyword heading word, floating subheading word, candidate term word]

14. phone app*.mp. [mp=title, abstract, heading word, drug trade name, original title, device manufacturer, drug manufacturer, device trade name, keyword heading word, floating subheading word, candidate term word]

**Young People**

15. Adolescent/ or young people.mp. or Child/

16. young pe*.mp. [mp=title, abstract, heading word, drug trade name, original title, device manufacturer, drug manufacturer, device trade name, keyword heading word, floating subheading word, candidate term word]

17. adole*.mp. [mp=title, abstract, heading word, drug trade name, original title, device manufacturer, drug manufacturer, device trade name, keyword heading word, floating subheading word, candidate term word]

18. child*.mp. [mp=title, abstract, heading word, drug trade name, original title, device manufacturer, drug manufacturer, device trade name, keyword heading word, floating subheading word, candidate term word]

19. student.mp. or Students/

**Further Mental Health Identifiers**

20. mental health.mp. [mp=title, abstract, heading word, drug trade name, original title, device manufacturer, drug manufacturer, device trade name, keyword heading word, floating subheading word, candidate term word]

21. mental disorder*.mp. [mp=title, abstract, heading word, drug trade name, original title, device manufacturer, drug manufacturer, device trade name, keyword heading word, floating subheading word, candidate term word]

22. psych*.mp. [mp=title, abstract, heading word, drug trade name, original title, device manufacturer, drug manufacturer, device trade name, keyword heading word, floating subheading word, candidate term word]

**Engagement and Effectiveness**

23. Attitude/ or attitude.mp.

24. response.mp.

25. experienc*.mp.

26. engagement.mp.

**Ethnicity**

27. ethnic*.mp. or exp "Ethnic and Racial Minorities"/ or exp Ethnicity/

**Term combination**

28. 23 or 24 or 25 or 26

29. 1 or 20 or 21 or 22

30. 15 or 16 or 17 or 18 or 19

31. 2 or 3 or 4 or 5 or 6 or 7 or 8 or 9 or 10 or 11 or 12 or 13 or 14

32. 27 and 28 and 29 and 30 and 31
